# Addition to “High On/Off Ratio Carbon Quantum Dot–Chitosan Biomemristors with Coplanar Nanogap Electrodes”

**DOI:** 10.1021/acsaelm.3c00153

**Published:** 2023-02-15

**Authors:** Niloufar Raeis-Hosseini, Dimitra G. Georgiadou, Christos Papavassiliou

**Affiliations:** † Department of Electronics and Electrical Engineering, Imperial College London, London SW7 2BT, United Kingdom; § Electronics and Computer Science, University of Southampton, Southampton SO17 1BJ, United Kingdom

In the original version of the article, the first author, who is the corresponding author, is adding her current address. The Acknowledgment has been corrected and is given below.

